# On the identity of *Liolaemus nigromaculatus* Wiegmann, 1834 (Iguania, Liolaemidae) and correction of its type locality

**DOI:** 10.3897/zookeys.294.4399

**Published:** 2013-04-22

**Authors:** Jaime Troncoso-Palacios, Carlos F. Garin

**Affiliations:** 1 Programa de Fisiología y Biofísica, Facultad de Medicina, Universidad de Chile, Casilla 70005, Santiago, Chile; 2 Departamento de Ecología, Facultad de Ciencias Biológicas, Pontificia Universidad Católica de Chile, Casilla 114-D, Santiago, Chile

**Keywords:** *Liolaemus*, *nigromaculatus*, *bisignatus*, *copiapoensis*, Atacama

## Abstract

In the current study, we review the taxonomic status of *Liolaemus nigromaculatus*. Despite being the nominal species of the *nigromaculatus* group and being the second species of the genus *Liolaemus* that was described, this species is of uncertain type locality and its true identification is a matter of discussion. After carefully analyzing several digital pictures of the holotype (juvenile male), reviewing all of the literature concerning the issue, examining specimens of nearly all recognized species of the *nigromaculatus* group, and determining the locations visited by the specimen collector, we are able to point out the following: 1) *Liolaemus nigromaculatus* was collected between Puerto Viejo and Copiapó of the Atacama region in Chile, and not in Huasco 2) *Liolaemus bisignatus* is a *nomen nudum*, and populations attributed to *Liolaemus bisignatus* should be referred to as *Liolaemus nigromaculatus*. 3) There is agreement that *Liolaemus copiapoensis* is indistinguishable from populations currently referred to as *Liolaemus bisignatus* (= *Liolaemus nigromaculatus*), 4) Populations found in Huasco (currently considered the type locality of *Liolaemus nigromaculatus*) are very similar to those found in Caldera (currently considered *Liolaemus bisignatus*) and should be designated as *Liolaemus nigromaculatus*, and 5) *Liolaemus oxycephalus* and *Liolaemus inconspicuus* are not synonymous with *Liolaemus nigromaculatus*, although their true identities are difficult to determine. We also detail several characteristic based on the holotype of *Liolaemus nigromaculatus*, in addition to drawing diagnostic comparisons between this species and others belonging to the *nigromaculatus* group.

## Introduction

The genus *Liolaemus* is comprised of 230 species (Uetz 2012) distributed throughout the southern portion of South America from the central mountains of Peru to the Tierra del Fuego in Chile. *Liolaemus nigromaculatus* (Wiegmann 1834) belongs to the subgenus *Liolaemus* and the *nigromaculatus* group (Lobo 2005). This is the second species that was described for the genus *Liolaemus* and it is the nominal species of the *nigromaculatus* group. However, *Liolaemus nigromaculatus* is a species with an uncertain provenance and a muddled taxonomic history (Donoso-Barros 1966, Valladares 2011). Apart from the original description, only Müller and Hellmich (1933a) have indicated data for this species based on the holotype, while the latest revisions either do not indicate the material examined (Ortiz 1981) or the specimens examined were not deposited in an institutional collection (Pincheira-Donoso and Núñez 2005).

Wiegmann (1834) described *Tropidurus nigromaculatus* from Chile based on one juvenile specimen collected by Franz Julius Ferdinand Meyen on his journey around the world during 1830-1832, without making mention of a specific type locality. He pointed out that the species is characterized by a gray color and rhomboid-oval shaped dorsal scales which have a keel but are obtuse. Also, he indicates that the scales of the dorsum are black spotted, “sind die einzelnen schuppen am Grunde schwarz…” (231 p), and that the dorsal pattern presents two series of dark spots which transversely extend to the base of the tail.

**Figure 1.**
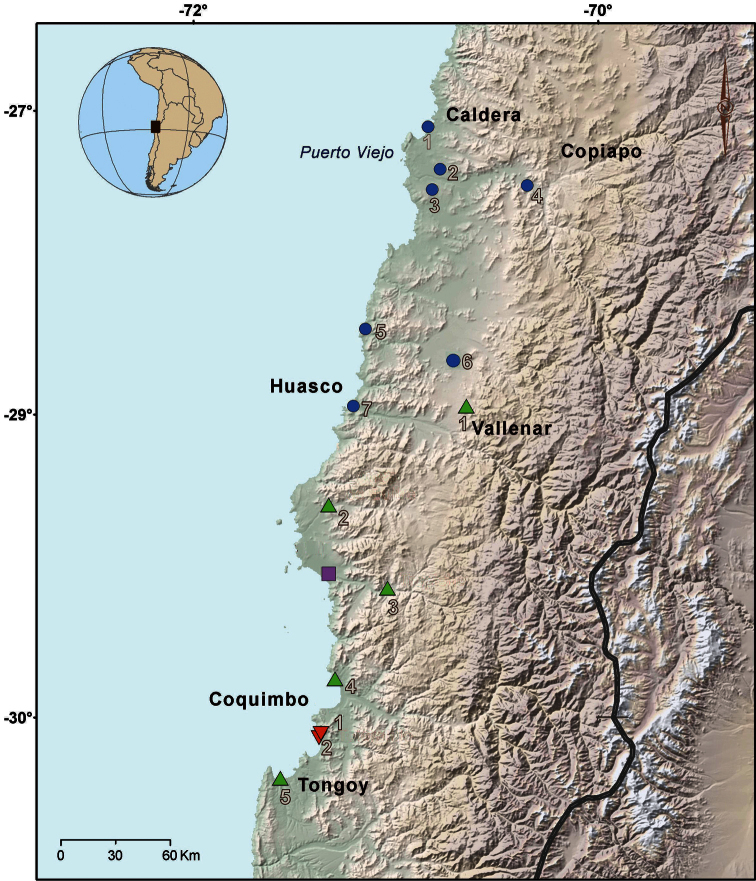
Distribution map of specimens used in this study. Blue circle: *Liolaemus nigromaculatus* (**1** Caldera **2** Road between Caldera and Copiapó **3** Near to Puerto Viejo **4** Copiapó **5** Llanos de Challe **6** Road between Vallenar and Copiapó and **7** Huasco). Green triangle: *Liolaemus atacamensis* (**1** Near to Vallenar **2** Lomas de Buitre **3** El Trapiche **4** Punta Teatinos and 5 Pachingo). Lilac square: *Liolaemus silvai* (Carrizalillo). Red triangle: *Liolaemus zapallarensis* (**1** Totoralillo and **2** Las Tacas). The main localities are indicated.

Duméril and Bibron (1837) transferred the species to the genus *Proctotretus* and provided a re-description. They show uncertainty about the locality of origin for *Proctotretus nigromaculatus*, stating that they examined specimens from Coquimbo (Fig. 1), which were collected by Charles Gaudichaud and deposited in the Muséum National d’Histoire Naturelle, France. These authors mention that the species has large scales which are strongly keeled and mucronate on the dorsum and flanks, characteristics that do not match with the description made by Wiegmann (1834).

Later, Fitzinger (1843) created the genus *Ptychodeira* and designated *Ptychodeira nigromaculata* as its type species. The author indicates Chile as the type locality. Although he does not list the specimens examined, he indicates that they are located in the Muséum National d’Histoire Naturelle, France.

Bell (1843) examined one specimen collected by Charles Darwin in Coquimbo and once again included the species in the genus *Proctotretus*. He points out that the dorsal scales of this species are strongly keeled and with mucrons.

Gray (1845) considered this species a member of the genus *Liolaemus*, based on specimens from Coquimbo. The description of the species is very similar to that made by Duméril and Bibron (1837) and by Bell (1843). Later, Girard (1858a, b) included the species within the genus *Rhytidodeira*, a decision that was rejected by Steindachner (1867), who once again included the species in the genus *Liolaemus*.

Boulenger (1885), examined three specimens from Coquimbo and three specimens from unknown origins, all deposited in the British Museum (London, England), and described them similar to those mentioned above for specimens from Coquimbo. Also, he indicated that *Liolaemus oxycephalus* (Wiegmann 1834), *Liolaemus inconspicuus* (Gray 1845), and *Liolaemus pallidus* (Philippi 1860) are synonyms of *Liolaemus nigromaculatus*, although he did not provide data to support these claims.

With such uncertainty about the type locality, Müller and Hellmich (1933a) finally examined the holotype deposited in Museum für Naturkunde (Berlin, Germany), stating that the holotype of *Tropidurus nigromaculatus* is numbered “ZMB 613” and suggesting that the true type locality of this species is Huasco (Fig. 1), as specimens from this locality are those that most resemble the holotype of *Liolaemus nigromaculatus*: *Am besten stimmt mit dem typus von n. nigromaculatus ein exemplar unseres materials überein, das von Huasco stammt* (128 p).

Unfortunately, Müller and Hellmich (1933a) did not describe the characteristics that led to these conclusions. Also, they indicate that the only known specimen is a juvenile (Müller and Hellmich 1933b). Additionally, these authors described several subspecies of *Liolaemus nigromaculatus* (*Liolaemus nigromaculatus atacamensis*, *Liolaemus nigromaculatus ater*, *Liolaemus nigromaculatus copiapoensis*, *Liolaemus nigromaculatus kuhlmanni*, and *Liolaemus nigromaculatus zapallarensis*) and included *Liolaemus bisignatus* (Philippi 1860) as a subspecies of *Liolaemus nigromaculatus* (Müller and Hellmich 1933a, b).

Donoso-Barros (1954) described a new subspecies, *Liolaemus nigromaculatus sieversi*. Thereafter, in his classic book Reptiles de Chile, Donoso-Barros (1966) states that it “has been very difficult to establish with certainty the status of the *Liolaemus nigromaculatus nigromaculatus* subspecies, initially described by Wiegmann. Müller and Hellmich considered Huasco as the type locality” (our translations). Faced with this uncertainty, Donoso-Barros (1966) decided to transcribe part of the original description and present it in his book.

Ortiz (1981) performed an analysis of the subspecies of *Liolaemus nigromaculatus* and concluded that *Liolaemus nigromaculatus sieversi* and *Liolaemus nigromaculatus ater* are subspecies of *Liolaemus zapallarensis*, and that *Liolaemus bisignatus*, *Liolaemus copiapoensis*, *Liolaemus kuhlmanni*, and *Liolaemus zapallarensis* are full species, so *Liolaemus nigromaculatus* included no subspecies. Unfortunately, he did not include *Liolaemus atacamensis* in his analysis. Although Ortiz (1981) indicated several characteristics for *Liolaemus nigromaculatus*, he did not list the specimens examined and only lists the localities, which are from Huasco to southern Coquimbo. However, Troncoso and Ortiz (1987) list specimens of *Liolaemus nigromaculatus* located in the Museo Regional de Concepción, Chile.

Simonetti and Núñez (1986) recorded specimens of *Liolaemus nigromaculatus* from Sierra Las Tapias, to the north of Chañaral. They pointed out that *Liolaemus nigromaculatus* can be differentiated from *Liolaemus atacamensis*, but they did not included a comparison with *Liolaemus bisignatus*.

Finally, Pincheira-Donoso and Núñez (2005), in a review of the Chilean species of the genus *Liolaemus*, redescribe *Liolaemus nigromaculatus*, keeping Huasco as the type locality. However, the authors examined specimens from Pan de Azúcar, Diego de Almagro, and Inca de Oro, localities never before mentioned for this species. In addition, these specimens were not deposited in a formal collection and were instead incorporated in the personal collection of D. Pincheira-Donoso, which is not located in any public or private institution, and so their results are currently unverifiable.

Therefore, in this paper we review the taxonomic status of *Liolaemus nigromaculatus* through the characterization of the holotype and clarification of the location in which it was collected. In addition, we provide a diagnosis respect of the other species of the *nigromaculaus* group.

## Materials and methods

The characteristics used for descriptions were taken from Ortiz (1981), Etheridge (1995), and Lobo (2001, 2005). Body measurements were taken with a digital vernier caliper (0.02 mm precision). Observations of scales were performed under different magnifying lenses. Through the courtesy of Frank Tillack (Museum für Naturkunde, Berlin, Germany), we examined high-resolution pictures from several views of the holotypes of *Liolaemus nigromaculatus* (ZMB 613) and *Liolaemus oxycephalus* (ZMB 615). Measurements and midbody scale counts for the ZMB 613 specimen were taken from Müller and Hellmich (1933a). Finally, some specimens were collected with a noose in several locations of the Coquimbo and Atacama Region, Chile: El Trapiche, Lomas de Buitre, Caldera and near to Puerto Viejo (Fig. 1). These specimens were fixed in 95% ethanol, preserved in 70% ethanol, and were deposited in Colección de Flora y Fauna, Profesor Patricio Sánchez Reyes of the Pontificia Universidad Católica de Chile (SSUC Re). These and other specimens examined are listed in Appendix I. We performed a Student’s *t* -test for comparison of SVL between *Liolaemus bisignatus* and *Liolaemus copiapoensis*. Data for *Liolaemus ater* was taken from Donoso-Barros (1966).

Acronyms mentioned in this publication are: MNHN-CL (Museo Nacional de Historia Natural, Chile), MZUC (Museo de Zoología, Universidad de Concepción), MRC (Museo Regional de Concepción), SSUC Re (Colección de Flora y Fauna Patricio Sánchez Reyes, Pontificia Universidad Católica de Chile) and ZMB (Museum für Naturkunde).

## Results

**Figure 2.**
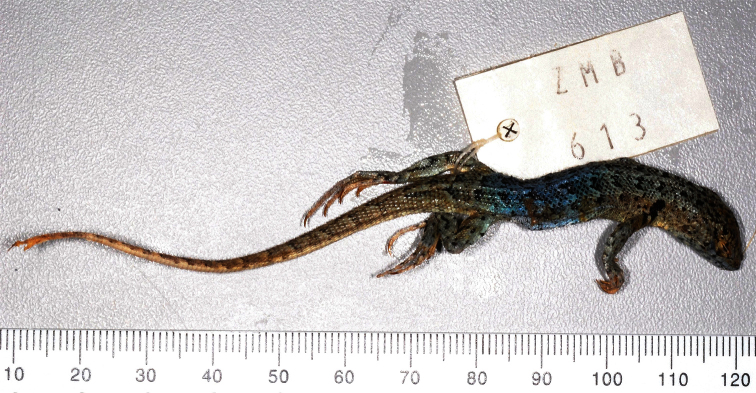
Dorsal view of the *Liolaemus nigromaculatus* holotype (ZMB 613).

**Figure 3.**
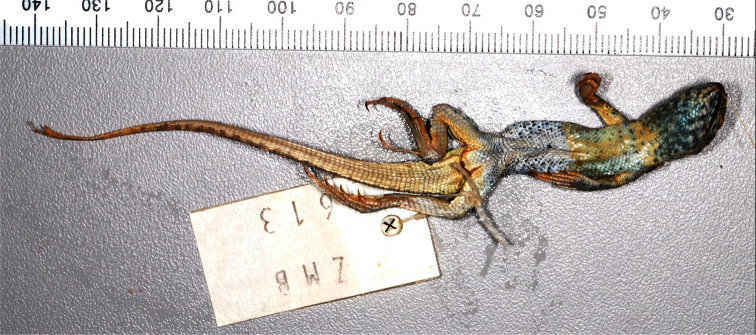
Ventral view of the *Liolaemus nigromaculatus* holotype (ZMB 613). Photograph by F. Tillack.

**Characteristics of the *Liolaemus nigromaculatus* holotype, ZMB 613.** The holotype is a juvenile male (Figs 2, 3). The following measurements were taken from Müller and Hellmich (1933a): SVL = 48 mm; Tail length = 76 mm; Head length = 12 mm; Head width = 10 mm; Head height = 7 mm; Forelimb length = 20 mm; Hindlimb length = 33 mm; and Midbody scales = 53. Furthermore, we observed the following: Pentagonal interparietal is smaller than the parietals and surrounded by six scales; seven scales between the interparietal and rostral; orbital semicircles are incomplete; four supraoculars; six supercilliaries scales and projected ciliary scales. The subocular is whitish and with a vertical black line at the center. The frontal region is fragmented into four scales. There are five scales between the frontal region and rostral scale; two scales between the nasal and canthal. The nasal is separated from the rostral by one scale and surrounded by five scales. One row of lorilabials between the supralabials and subocular; four supralabials, with the fourth curved upward and without contacting the subocular. Five infralabial scales and four pairs of post-mental shields with the second pair being in contact. Two scales on the anterior edge of the ear, projected onto the meatus but without covering it. Temporal scales are smooth (a few are slightly keeled) and subimbricated. The lateral neck fold is “Y” shaped and an antehumeral fold is present. Six temporal scales between the level of the supercilliaries and commissure. Dorsal scales are rounded or lanceolated, imbricated, slightly keeled, and without mucrons. Ventral scales are rounded, smooth, and subimbricated. There are at least 80 ventral scales and three precloacal pores, two according to Müller and Hellmich (1933a). Dorsal scales of the tail are rounded, imbricated, keeled, and mucronate.

**Figure 4.**
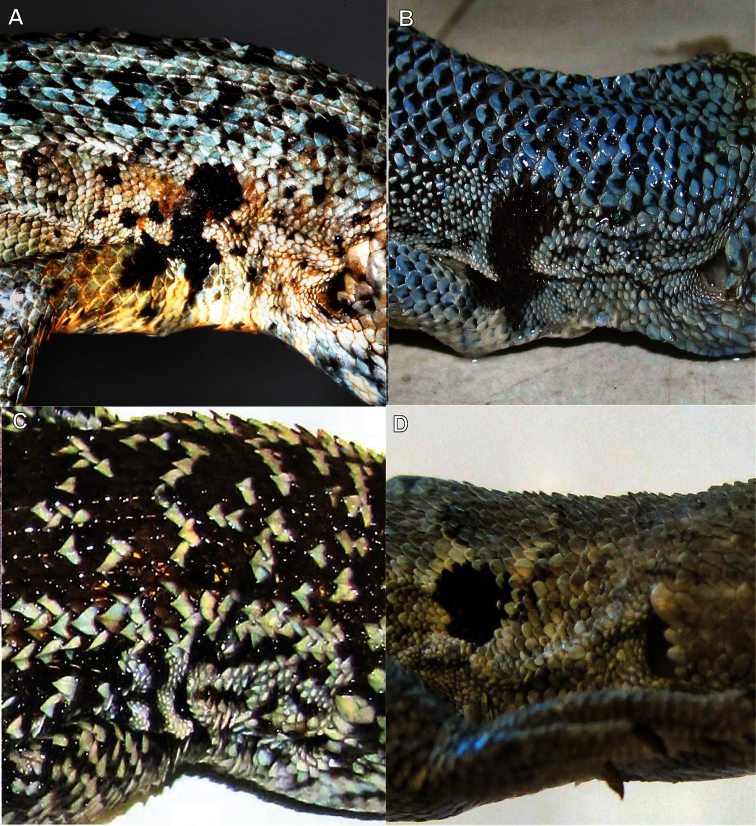
Lateral shoulder view of some specimens studied. **A** Holotype of *Liolaemus nigromaculatus* (ZMB 613, juvenile male) with black “ג” shaped antehumeral spot and dorsal scales without mucrons (Photograph by F. Tillack) **B** Holotype of *Liolaemus bisignatus* (=*Liolaemus nigromaculatus*, adult male MNHN-CL 1477) with black “ג” shaped antehumeral spot and dorsal scales without mucrons **C** Male specimen of *Liolaemus zapallarensis* (MZUC 29118) with irregular antehumeral spot and dorsal scales with mucrons **D** Male specimen of *Liolaemus atacamensis* (SSUC Re 469) with rounded antehumeral spot and dorsal scales without mucrons.

**Color in alcohol**. Dorsal and lateral views of the head are light brown in color, the same tone of the dorsum, and have numerous black spots which do not form a clear pattern. On the neck, these spots become smaller. The color of the dorsum is gray-brown (gray according to Wiegmann 1834). Nine series of dark spots are over the paravertebral fields, from the base of the neck to the base of the tail. These spots are composed of approximately 8-10 scales. Additionally, over the dorsum there are numerous black spotted scales. The temporal band has seven dark spots, which are smaller than the spots over the paravertebral fields. There is a marked, black antehumeral spot from the shoulder to the humeral zone which shows a constriction in the middle and is divided into two at the base, as forming a “ג” shape (Fig. 4). Forelimbs and hindlimbs have a gray-brown color and black spots. Flanks are of a gray-brown color. The tail has a brown color and few dark spots. The belly, ventral surface of the tail, and ventral surface of forelimbs and hindlimbs are whitish. The belly has abundant dark spots. The throat has a strong dark reticulation.

**Diagnosis of the *nigromaculatus* group.** Currently, it is difficult to establish a diagnosis for this group. Preliminary evidence in an ongoing molecular phylogenetic study (Troncoso-Palacios and Schulte, *in prep*) shows that this group is composed of two clades. One clade includes *Liolaemus atacamensis*, *Liolaemus ater*, *Liolaemus kuhlmanni*, *Liolaemus melaniceps*, *Liolaemus nigromaculatus*, *Liolaemus sieversi*, *Liolaemus silvai* and *Liolaemus zapallarensis*; and the second includes *Liolaemus hellmichi*, *Liolaemus platei* and *Liolaemus velosoi*. A similar proposal is made in Pincheira-Donoso and Núñez (2005). The first clade (*nigromaculatus* group, *sensu stricto*) can be distinguished from other groups of the *Liolaemus* subgenus through the following combination of characteristics: 1) nasal and rostral scales separated by one scale, 2) an antehumeral black spot whether it is on males, females or juveniles, and 3) a series of black spots on the paravertebral fields at least in juveniles.

However, since a formal study is lacking, the following diagnosis includes the currently considered species as members of the group *nigromaculatus*.

**Diagnosis of *Liolaemus nigromaculatus* based on the holotype.** *Liolaemus nigromaculatus* can be differentiated from the other species of the *nigromaculatus* group through the following combination of characteristics: 1) nasal and rostral scales separated by one scale, 2) presence of projected ciliary scales, 3) dorsum with abundant, black-spotted scales, 4) series of black spots on the paravertebral fields, from the base of the neck to the base of the tail, 5) marked “ג” shaped antehumeral black spot from the shoulder to the humeral zone, 6) dorsal scales are rounded or lanceolated, slightly keeled, and without mucrons. Diagnosis with respect to other species of the *nigromaculatus* group is listed below.

*Liolaemus nigromaculatus* differs from *Liolaemus hellmichi* (Donoso-Barros 1975), *Liolaemus platei* (Werner 1898), and *Liolaemus velosoi* (Ortiz 1987) because in all of these species the nasal scale always contacts the rostral scale and they never have projected ciliary scales.

*Liolaemus nigromaculatus* differs from *Liolaemus melaniceps* (Pincheira-Donoso and Núñez 2005) because in this species the dorsal scales are juxtaposed. In contrast, *Liolaemus nigromaculatus* has imbricated dorsal scales. Head color in *Liolaemus melaniceps* is remarkably darker than the body, whereas *Liolaemus nigromaculatus* maintains the same color.

*Liolaemus nigromaculatus* differs from *Liolaemus ater*, *Liolaemus kuhlmanni*, *Liolaemus zapallarensis* (Müller and Hellmich 1933a, b), *Liolaemus sieversi* (Donoso-Barros 1954) and *Liolaemus silvai* (Ortiz 1989)because these species have strongly keeled and mucronate dorsal scales (Fig. 4), whereas in *Liolaemus nigromaculatus* dorsal scalesare not mucronate and are slightly keeled.

Finally, *Liolaemus nigromaculatus* differs from its most similar species, *Liolaemus atacamensis* (Müller and Hellmich 1933b), because this latter species never has black spotted scales on the dorsum, which are abundant in *Liolaemus nigromaculatus*. The antehumeral spot is rounded in male *Liolaemus atacamensis* and does not contact the humeral zone (Fig. 4, Table 1). Moreover, the male *Liolaemus atacamensis* has abundant blue-sky scales dispersed over the dorsum, a trait absent in *Liolaemus nigromaculatus*. The ventral scales vary between 66-77 for *Liolaemus atacamensis* but are at least 80 in *Liolaemus nigromaculatus*.

**Table 1.** Scale and morphological characteristics of geographically close species to *Liolaemus nigromaculatus* (examined juveniles are excluded). M = males and F = females.<br/>

	*Liolaemus nigromaculatus* (Puerto Viejo - Copiapó and surroundings) <br/>M=19, F=16	*Liolaemus atacamensis* <br/>M=7, F=9	*Liolaemus kuhlmanni* <br/>M=7, F=9	*Liolaemus zapallarensis* <br/>M=1, F=2
Midbody Scales	50–61	48–54	52–58	48–52
Ventrals	77–84	66–77	80–90	79–80
Nasal Separated from Rostral	Yes	Yes	Yes	Yes
Color of Male	Gray (with olive or yellow shades)	Gray or brown	Black	Black
Shape of Dorsal Scales	Rounded or lanceolate	Rounded or lanceolate	Lanceolate	Lanceolate
Mucrons in Dorsal Scales	No	No	Yes	Yes
Black Spotted Dorsal Scales	Yes	No	No	No
Male with “ג” Shaped Antehumeral Spot	Yes	No	Indistinguishable	Indistinguishable
Maximum SVL (mm)	83.0	67.2	81.2	72.4

**The relationship between *Liolaemus bisignatus* (Philippi 1860) and *Liolaemus copiapoensis* (Müller and Hellmich 1933b).** Philippi (1860) illustrated (without a description, see below) *Proctotetrus bisignatus* (= *Liolaemus bisignatus*) without mention of a type locality. Later, Müller and Hellmich (1933b) restricted the type locality of *Liolaemus bisignatus* to Caldera, Chile. On the other hand, Müller and Hellmich (1933b) described *Liolaemus nigromaculatus copiapoensis* as being from Copiapó. Hellmich (1950) indicated that *Liolaemus nigromaculatus copiapoensis* is very similar to *Liolaemus nigromaculatus bisignatus*, and differs in being smaller and lacking a light green color. Donoso-Barros (1966) also noted the similarities between *Liolaemus nigromaculatus copiaponesis* and *Liolaemus nigromaculatus bisignatus*, although he considered both species valid and added as a diagnosing characteristic the absence of a keel on temporal scales of *Liolaemus nigromaculatus copiapoensis*. Conversely, Ortiz (1981) states that the two species do not differ in the size or development of a keel on the temporal scales. Although in his analysis both species appear to be very close, he points out the following diagnostic characteristics: the ventral color of the thighs and cloaca is yellow in *Liolaemus bisignatus* and orange in *Liolaemus copiapoensis* (listed as a weak difference), *Liolaemus bisignatus* has dorsal color grayish green whereas *Liolaemus copiapoensis* has dorsal color yellowish white, *Liolaemus bisignatus* is a coastal species while *Liolaemus copiapoensis* is a valley species, and *Liolaemus bisignatus* takes refuge in dunes whereas *Liolaemus copiapoensis* does so in burrows. Lobo (2001, 2005), in a phylogenetic analysis, found both species to be sister taxa, but maintains their status as full species.

Pincheira-Donoso and Núñez (2005), after studying topotypes of both species, concluded that *Liolaemus copiapoensis* is a synonym of *Liolaemus bisignatus*. However, Valladares (2011) considered *Liolaemus copiapoensis* a valid species.

We agree with Pincheira-Donoso and Núñez (2005) as our examination of topotypes of both populations, including the specimen considered as the holotype of *Liolaemus bisignatus*, shows that: 1) Both species do not differ in size. Adult males of *Liolaemus bisignatus* (n = 11; = 74.1 mm; rank = 60.9 – 83.0 mm) do not show significant differences as compared to adult males of *Liolaemus copiapoensis* (n = 8; = 70.1 mm; rank = 60.7 – 78.1 mm) (*t* = 1.27; *P* = 0.22). Adult females of *Liolaemus bisignatus* (n = 12; = 63.7 mm; rank = 56.6 – 80.7 mm) do not show significant differences compared to adult females of *Liolaemus copiapoensis* (n = 4; = 56.9 mm; rank = 56.5 – 59.8 mm) (*t* = 1.69; *P* = 0.11), 2) Both species do not differ in color pattern, as the males of *Liolaemus bisignatus* can have orange color on thighs and cloaca and males of both populations have a gray dorsal color, with green or yellow shades in some specimens, 3) Both species have smooth or slightly keeled temporal scales, whith keel more developed in males, 4) The distribution of populations attributable to *Liolaemus bisignatus* or *Liolaemus copiapoensis* is continuous from the coast to the valley, and the type of refuge used by these lizards cannot be used to identify a species as this depends on the availability of refuge types in the habitat.

**The relationship between *Liolaemus nigromaculatus* (Wiegmann 1834) and *Liolaemus bisignatus* (Philippi 1860).** Philippi (1860), included eight species of reptiles and one amphibian (all numbered) in the “Zoology of the Atacama Desert” section of his book. Among them, *Proctotretus nigromaculatus* (= *Liolaemus nigromaculatus*, number 2) was briefly mentioned and he pointed out that in the lamina of his book the species is labeled as *Proctotretus bisignatus*: “Tab. VI, Fig. 2, nomine *Proct. bisignatus*”. Apparently, Philippi (1860) intended to describe the specimen that he collected as a new species (*Proctotretus bisignatus*), but subsequent to the completion of the lamina, he would have realized that the species was already described as *Proctotretus nigromaculatus*. In fact, Philippi (1860) only provides three data for *Proctotretus nigromaculatus*: snout-vent length (SVL), tail length, and shape of dorsal scales. Therefore, *Liolaemus bisignatus* was never described by Philippi (1860). Indeed, according to Article 12.1 of the “Names published before 1931” section of the International Code of Zoological Nomenclature, *Liolaemus bisignatus* is a *nomen nudum* because it was never described, as to be available every new name published before 1931 must be accompanied by a description or a definition of the taxon that it denotes, or by an indication (ICZN 1999).

**Figure 5.**
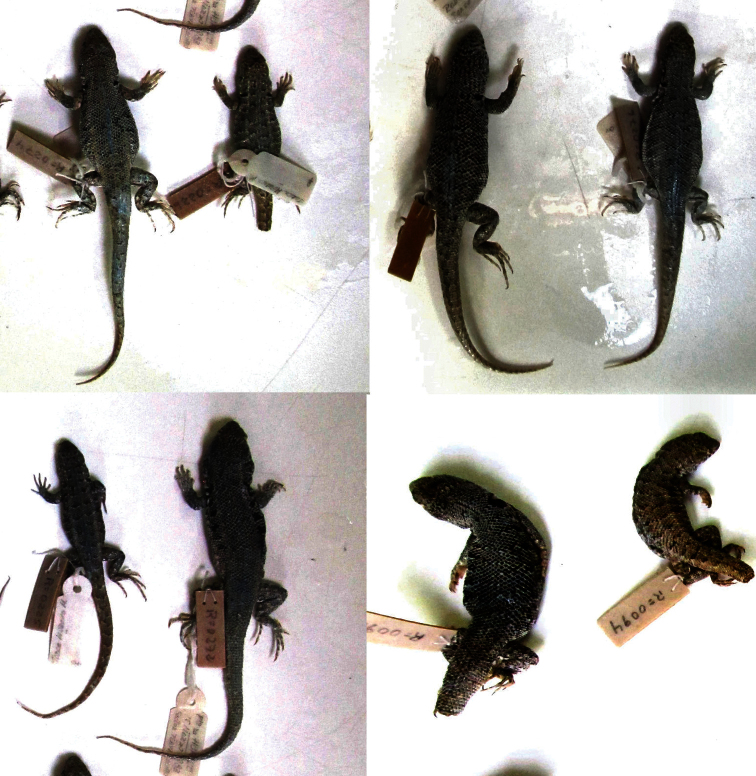
Some specimens listed by Troncoso and Ortiz (1987). All are assignable to *Liolaemus nigromaculatus***,** from top left to bottom right: MRC 274 and 226 (*Liolaemus nigromaculatus* from Huasco), MRC 282 (*Liolaemus nigromaculatus* from Caldera), MRC 276 (*Liolaemus bisignatus* from Caldera), MRC 225 (*Liolaemus nigromaculatus* from Huasco), MRC 272 (*Liolaemus bisignatus* from Huasco), and MRC 090 and 094 (*Liolaemus copiapoensis* from Copiapó).

The second publication which deals with this species (Müller and Hellmich 1933b) includes *Liolaemus bisignatus* as a subspecies of *Liolaemus nigromaculatus*. However, Müller and Hellmich (1933b) indicated that it is probable *Liolaemus bisignatus* could be a synonym of *Liolaemus nigromaculatus*. Later, Ortiz (1981) considered *Liolaemus bisignatus* a full species, a status which remains until today. Although Ortiz (1981) did not list the specimens examined, Troncoso and Ortiz (1987) list several specimens of *Liolaemus nigromaculatus* (from Huasco and Caldera) and *Liolaemus bisignatus* (from Huasco and Caldera). This mixture of locations suggests a difficulty in differentiating both species. Our examination of these specimens shows that all are assignable to *Liolaemus nigromaculatus* (Fig. 5). Although Philippi (1860) did not designate a holotype or type locality for *Liolaemus bisignatus*, Müller and Hellmich (1933b) restrict the type locality to Caldera, Chile, and according to Ortiz and Núñez (1986), the holotype is specimen MNHN-CL 1477 collected by R.A. Philippi in Atacama.

**Figure 6.**
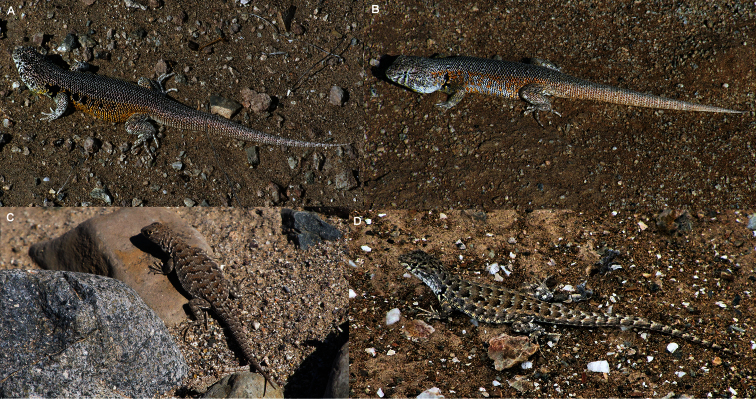
Variation of color pattern in *Liolaemus nigromaculatus*. **A** and **B** Adult males from 20 Km SE from Puerto Viejo **C** Adult female from the same location **D** Juvenile from Caldera.

**Figure 7.**
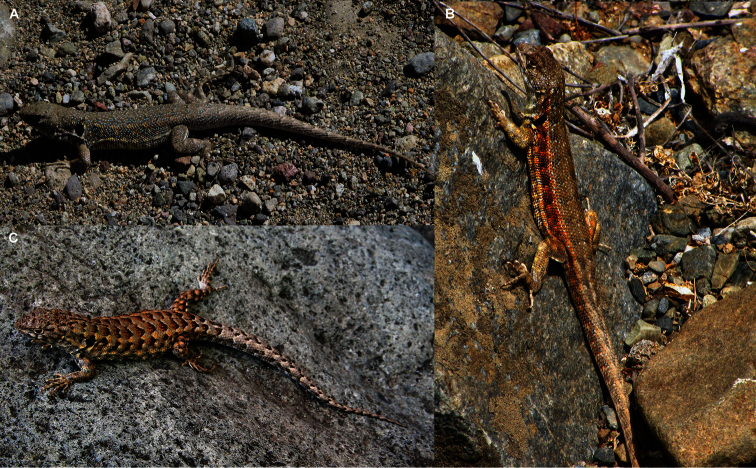
Variation of color pattern in *Liolaemus atacamensis*. **A** Adult male from El Trapiche **B** Adult male from Lomas de Buitre **C** Adult female from Lomas de Buitre.

Our review of twenty-three specimens of *Liolaemus bisignatus* (adults and juveniles, including the holotype) allows us to determine that the populations currently referred to as *Liolaemus bisignatus* should be referred to as *Liolaemus nigromaculatus* based on the following: 1) Of the species in the *nigromaculatus* group that have the nasal separated from the rostral, only *Liolaemus bisignatus* and *Liolaemus atacamensis* (Figs 6, 7) have dorsal scales without mucrons, and of these, only *Liolaemus bisignatus* overlaps with the diagnostic characters of *Liolaemus nigromaculatus*, 2) The color of the *Liolaemus nigromaculatus* holotype is brown-gray with a series of black spots over the paravertebral fields, as in juveniles of *Liolaemus bisignatus*, 3) *Liolaemus nigromaculatus* has a black“ג” shaped antehumeral spot, from the shoulder to the humeral zone, like *Liolaemus bisignatus*, 5) *Liolaemus nigromaculatus* has abundant black spotted scales on the dorsum, like *Liolaemus bisignatus*, 6) The holotype of *Liolaemus nigromaculatus* has at least 80 ventral scales, which is in the range of *Liolaemus bisignatus* but not for *Liolaemus atacamensis* (Table 1), 7) Of the species in the *nigromaculatus* group with the nasal separated from the rostral, only *Liolaemus bisignatus* and *Liolaemus atacamensis* are known to be from the zone in which Meyen collected the holotype of *Liolaemus nigromaculatus* (see below), and of these only *Liolaemus bisignatus* overlaps with the diagnostic characteristics of *Liolaemus nigromaculatus*, 8) In Huasco, the location currently accepted as the type locality of *Liolaemus nigromaculatus*, it is only possible to find two other species of *Liolaemus* (*Liolaemus bisignatus* and *Liolaemus fuscus*), this explains why Müller and Hellmich (1933a) assignedHuasco as the type locality of *Liolaemus nigromaculatus*. In contrast to *Liolaemus nigromaculatus*, *Liolaemus fuscus* has the nasal scale always in contact with the rostral.

**The true type locality of *Liolaemus nigromaculatus*.** Wiegmann (1834) described *Liolaemus nigromaculatus* based on one specimen collected by the doctor and naturalist FJF Meyen. Wiegmann (1834) designated “Chile” as the type locality. Later, Duméril and Bibron (1837) indicated that *Liolaemus nigromaculatus* inhabits Coquimbo because the examined specimens from this locality were, according to their criteria, consistent with the description of Wiegmann (1834). Thereafter, Müller and Hellmich (1933a) reviewed the holotype and proposed Huasco as the type locality based on the similarities of *Liolaemus nigromaculatus* with the examined specimens from Huasco. For this reason, the subsequent studies that have mentioned this species consider Huasco as the type locality (Donoso-Barros 1966, Ortiz 1981, Pincheira-Donoso and Núñez 2005).

According to Meyen (1834) he traveled for Chile for three months (from January to March 1831) mainly in the central region (San Fernando, Rancagua, Cajón del Maipo, Colina and Valparaíso). On March 6, he sailed from Valparaíso to Coquimbo where he stayed for two days. Later, he went to northern Chile, landing in Puerto Viejo (Puerto de Copiapó) on March 10 and then going to Copiapó and its surroundings (e.g. Tierra Amarilla, El Checo mine). Ten days later, he returned to Puerto Viejo and sailed to Peru. Meyen’s journey is also detailed in Domeyko (1859).

We can discard the area from San Fernando to Valparaíso as the type locality of *Liolaemus nigromaculatus* since no species of the *nigromaculatus* group has been documented for this area. On the other hand, all species of *nigromaculatus* group with the nasal scale separated from rostral and that inhabit the surroundings of Coquimbo (*Liolaemus atacamensis*, *Liolaemus kuhlmanni* and *Liolaemus zapallarensis*) are clearly distinguishable from *Liolaemus nigromaculatus*, so *Liolaemus nigromaculatus* is not found in Coquimbo.

Meyen never visited Huasco, so it is impossible that the specimen ZMB 613 was collected by him there. Müller and Hellmich (1933a) restricted the type locality of *Liolaemus nigromaculatus* to Huasco based on morphological characteristics, but without considering the probable location of collection. Later, this decision was never questioned by another author and the mistake remains today.

In conclusion, the true type locality of *Liolaemus nigromaculatus* should be restricted to the route and surroundings from Puerto Viejo to Copiapó because in the entirety of this route specimens of *Liolaemus nigromaculatus* can be found and are being by far the most abundant species. Since this route covers approximately 60 Km, it is impossible to obtain a more precise location.

**Variation of the species.** Variation analysis was based on 40 specimens (19 males, 16 females, and 5 juveniles) from a transect of Puerto Viejo – Copiapó and the surroundings, including Caldera. The data are as follows: SVL adult males: 75.7 ± 5.4 mm. SVL adult females: 67.4 ± 7.2 mm. Tail length in adult males: 84.4 ± 15.5 mm. Tail length in adult females: 80.8 ± 9.7 mm. Head length in adult males: 19.3 ± 1.8 mm. Head length in adult females: 17.6 ± 2.7 mm. Head width in adult males: 14.3 ± 0.9 mm. Head width in adult females: 11.9 ± 0.4 mm. Head height in adult males: 9.5 ± 1.0 mm. Head height in adult females: 8.0 ± 0.8 mm. Forelimb length in adult males: 25.4 ± 3.8 mm. Forelimb length adult in females: 19.6 ± 1.7 mm. Hind limb in adult males: 40.2 ± 5.5 mmm. Hind limb in adult females: 32.1 ± 1.6 mmm.

Pentagonal or hexagonal interparietal, smaller than the parietals and surrounded by 6-7 scales; 7-9 scales between the interparietal and rostral; orbital semicircles are complete, formed by 8-10 scales, or incomplete; 3-5 supraoculars and 6 supercilliaries. The subocular is whitish with a vertical black line at the center. Two scales between nasal and canthal. The nasal is separated from the rostral by one scale and surrounded by six scales. There is one row of lorilabials between the supralabials and subocular. There are 4-5 supralabials, the last of which is curved upward without contact to the subocular; 5-6 infralabial scales. The mental is pentagonal and in contact with four scales. There are four pairs of post-mental shields; the second pair is in contact or is separated by one scale. There are 2-3 enlarged scales on the anterior edge of the ear but without covering the meatus. Temporal scales are subimbricated and smooth or slightly keeled. The lateral neck fold is “Y” shaped and antehumeral fold is present. There are 6-7 temporal scales between the level of supercilliaries and commissure. Midbody scales: 50-61. Dorsal scales are rounded or lanceolated, subimbricated or imbricated, slightly keeled and without mucrons, and some specimens have interstitial granules. Dorsal scales are similar or smaller than the ventrals. Ventrals : 77-84. Ventral scales are rounded, smooth, subimbricate, and without interstitial granules. There are 2-3 precloacal pores in the males. The suprafemoral scales are rounded, imbricated, and keeled without mucrons. Infrafemoral scales are rounded, smooth, and imbricated. Supraantebrachials scales are rounded, imbricated, and keeled without mucrons. Infraantebrachials are rounded, imbricated, and smooth. The ventral tail scales are lanceolate, imbricated, keeled, and with mucrons.

Color: There is a highly variable color pattern, which varies with the size and sex of individuals. The juveniles have a brown head, with the same shade on the dorsum and with some dark spots or no spots. The dorsum has a brown-yellow color. The vertebral line is fragmented or absent, always disappearing in larger juveniles. There are nine to ten dark spots on the paravertebral fields, from the neck to the base of the tail. These spots have white or yellow on the posterior edge. There is a yellow supraocular line, and black “ג” shaped antehumeral spot. The temporal band is formed by four to seven dark spots which have white or yellow on the posterior edge. The limbs are brown with small black and white spots. There is a whitish ventral color, sometimes with small dark spots. The throat is striated or spotted. The tail is brown, with vertebral line and ringed.

For adult males, the following observations were made: The supraoculars lines of juveniles disappear. The head is gray, has the same shade as the body, and which, in some specimens, also has yellow or olive shades. The dark spots of the temporal bands and paravertebral fields progressively merge until disappearing in the larger specimens, leaving a gray color on the dorsum without design, and sometimes with olive and yellow shades. On the dorsum there are abundant black spotted scales, dispersed and without forming a design. There is a black “ג” shaped antehumeral spot, and in some specimens it is accompanied by three to six round and smaller dark spots from shoulder towards the rear. There is an absence of a vertebral line. In some specimens, the flanks have yellow or orange color. The tail has either a vertebral line or no design. The limbs are gray with small black and white spots, and the belly is whitish. The throat is striated or spotted, and the cloacal region is orange or yellow.

Finally, for females observations were as follows: There is a gray or brown color and abundant black spotted scales dispersed on the dorsum. There is a black “ג” shaped antehumeral spot, but it is more diffuse than in males. Females differ from the males in that they have dark spots over the dorsum with white on the posterior edge. Also, females have a yellow supraocular line. There is an absence of vertebral line. The limbs are gray with small black and white spots. There is a whitish belly, but in some specimens there is orange in the middle. The throat is striated or spotted. The tail has a vertebral line and may be ringed.

## Discussion

Without a doubt, one of the most confused issues in the taxonomy of Chilean herpetology is the definition of *Liolaemus nigromaculatus*, whose taxonomic status and type locality have been uncertain for many years (Donoso-Barros 1966, Troncoso-Palacios and Marambio 2011). Furthermore, the species is very polymorphic. The dorsal pattern varies with both size and sex. It is even possible that the yellow and orange color on the flanks of some adult males is related to the reproductive status. In the past, this led to the description of populations from Copiapó as a new species, *Liolaemus copiapoensis* (Müller and Hellmich 1933b). Moreover, some species have been placed under synonymy with *Liolaemus nigromaculatus* without appropriate justification. Boulenger (1885) indicated that *Liolaemus oxycephalus* (Wiegmann 1834) is a synonym of *Liolaemus nigromaculatus*. However, the holotype of *Liolaemus oxycephalus* has the nasal in contact with rostral scale, which is always separated in *Liolaemus nigromaculatus*. The type locality of *Liolaemus oxycephalus* is not indicated in its description, but the holotype of *Liolaemus oxycephalus* strongly resembles *Liolaemus platei* and *Liolaemus velosoi*. However, both of these species are very similar and inhabit localities visited by Meyen, and since the state of conservation of *Liolaemus oxycephalus* is poor, it is difficult to indicate a relationship of synonymy, so we propose maintaining the specific names of *Liolaemus platei* and *Liolaemus velosoi*. Boulenger (1885) also indicated that *Liolaemus pallidus* (Philippi 1860) from Paposo is a synonym of *Liolaemus nigromaculatus*. For the moment, it is impossible to clarify this issue because the types of *Liolaemus pallidus* are lost (Ortiz and Núñez 1986). Finally, Boulenger (1885) also indicated that *Liolaemus inconspicuus* (Gray 1845) is a synonym of *Liolaemus nigromaculatus*. The type locality of *Liolaemus inconspicuus* is not indicated in the description, but according to Gray (1845) *Liolaemus inconspicuus* has strongly keeled and mucronate dorsal scales, so it can not be a synonym of *Liolaemus nigromaculatus*.

Several authors mentioned Coquimbo as the inhabiting locality of *Liolaemus nigromaculatus* (Duméril and Bibron 1837, Bell 1843, Gray 1845, Boulenger 1885). However, these authors describe a lizard with strongly keeled and mucronated dorsal scales, and with a dorsal pattern formed by a series of dark spots. Probably, these authors confused juvenile specimens of *Liolaemus zapallarensis* or *Liolaemus kuhlmanni* with *Liolaemus nigromaculatus*.

The most similar species to *Liolaemus nigromaculatus* is *Liolaemus atacamensis*. Müller and Hellmich (1933b) described *Liolaemus atacamensis* from Atacama, north of Copiapó, based on two specimens (SVL = 57-55 mm). Later, Hellmich (1950) examined 18 more specimens from Vicuña and La Serena, both in Coquimbo Region, but unfortunately he does not provide SVL data, although he does provide the range of midbody scales: 48-54. We examined 16 specimens of *Liolaemus atacamensis* from several locations from both the Atacama and Coquimbo Regions, and the SVL (46.6 – 67.2 mm) and midbody scales (48-54) are in the range of previous data.

Use of digital pictures of type specimens has proved to be a powerful and useful tool for clarifying confusing taxonomic issues. Recently, Langstroth (2011) clarified the taxonomic status of *Liolaemus stolzmanni* (Steindachner 1891) and *Liolaemus pachecoi* (Laurent 1995), and Troncoso-Palacios and Etheridge (2012) restrict the distribution of *Liolaemus tacnae* (Shreve 1941) using digital pictures of types.

Here, we hope to have contributed to the clarification of the taxonomic identity of *Liolaemus nigromaculatus* in addition to providing new data and correcting some mistakes, all with the end of trying to understand the still uncertain semantics of the *nigromaculatus* group.

## Conclusion

The type locality of *Liolaemus nigromaculatus* should be restricted to the transect and surroundings of Puerto Viejo – Copiapó, and the populations currently recognized as *Liolaemus bisignatus* or *Liolaemus copiapoensis* are assignable to *Liolaemus nigromaculatus*.

## Appendix I

### Specimens examined

*Liolaemus atacamensis*. SSUC Re 469. 20 Km al norte de Vallenar. F. Ferri col. 2010. SSUC Re 470-71. El Trapiche, Coquimbo. J. Troncoso-Palacios, Y. Marambio & D. Hiriart cols. Mayo, 2012. SSUC Re 454, 464-68. Lomas de Buitre, Freirina, Atacama. J. Troncoso-Palacios, Y. Marambio & D. Hiriart cols. Mayo, 2012. SSUC Re 455-61. Playa Humedal Pachingo, entre Tongoy y Puerto Aldea, Coquimbo. C. Garin col. 10/12/2009. MZUC 30193, 30196. Punta Teatinos, sector de dunas costeras. J.C. Ortiz col. 14/09/1982.

*Liolaemus fuscus*. SSUC Re 258-59. Altos de Jahuel, Valparaíso. F. Ferri & J. Troncoso-Palacios. 19/12/2010. SSUC Re 255-57. Camino a Farellones. F. Ferri col. 18/12/2010. SSUC Re 260. Camino a Ovalle, Coquimbo. F. Ferri. 17/03/2012. MRC 229-34, 236. Huasco. J. Moreno col. May, 1983.

*Liolaemus hellmichi*. MZUC 25942-52. Cerro Moreno. J.C. Ortiz col. 02/04/2001.

*Liolaemus kuhlmanni*. MZUC 28829, 28838-41. Papudo. J. Simonetti & J.C. Ortiz col. 9/9/1977. SSUC Re 282-84. Dunas de Ritoque. F. Ferri col. 13/11/2010. SSUC Re 285-97. Caleta de La Ligua. F. Ferri col. 26/11/2010. SSUC Re 473. Dunas de Concón. J. Troncoso-Palacios & F. Urra cols. 04/01/2011.

*Liolaemus nigromaculatus*. SSUC Re 478 20 Km al sur este de Puerto Viejo. J. Troncoso-Palacios & Y. Marambio cols. Mayo, 2012. MRC 051-53. Caldera. J. Moreno col. Mayo, 1982. MRC 162, 273, 276, 282-83. Caldera. J. Moreno col. 1983. SSUC Re 306-15, 474-75. Caldera. F. Ferri col. Noviembre, 2011. SSUC Re 476-77. Caldera, Atacama. Y. Marambio col. Mayo, 2012. MNHN-CL 2237-38. 20 km de Caldera entre Copiapó y Caldera. H. Núñez col. 30/09/1991. SSUC Re 007. Llanos de Challe. G. Lobos, A. Channier & J. González cols. 2002. SSUC Re 453, 462. 50 Km al norte de Vallenar. J. Troncoso-Palacios col. 05/06/2011. MRC 087-94. Copiapó. C. Valdovinos col. 15/9/1984. MRC 514. Copiapó. Unknown collector. Noviembre, 1996. MNHN-CL 2249-55. Travesía, Copiapó. H. Núñez col. 28/09/1999. MRC 059. Huasco. J. Moreno col. 11/08/1982. MRC 225-28, 272, 274-75, 285. Huasco. J. Moreno col. 15/03/1983.

*Liolaemus melaniceps*. MNHN-CL 3601-03. Isla Chungungo. J.C. Torres-Mura col. January, 2002.

*Liolaemus platei*. MZUC 2152-53. Combarbalá. I. Peña col. Noviembre de 1961. SSUC Re 029. Llanos de Challe. G. Lobos, A. Channier & J. González cols. 2002. SSUC Re 316-20, 335-36, 380. Caldera. Francisco Ferri col. SSUC Re 321. Illapel. Francisco Ferri col. SSUC Re 420. Coquimbo. J. Troncoso-Palacios & Y. Marambio cols. 12/12/2011. SSUC Re 526. Coquimbo. J. Troncoso-Palacios & Y. Marambio cols. Mayo, 2012. MRC 058, 063. Chañaral. J. Moreno col. 28/7/1982.

*Liolaemus sieversi*. MZUC 8914, 12096-98, 12100, 12119-23, 12126, 12128. Isla Los Locos. R. Donoso-Barros col. 15/02/1954.

*Liolaemus silvai*. MZUC 30184. Carrizalillo. J.C. Ortiz col. February, 1983.

*Liolaemus velosoi*. MZUC 36612-14, 36618-20, 36624. Estación Paipote. J.C. Ortiz col. 16/02/1978. MZUC 32695, 32699, 32702, 32704, 32706. Copiapó. R. Moreno col. Febrero de 2000. MRC 054. Copiapó. Unknown collector. 16/06/1982. MRC 055. Copiapó. Unknown collector. 20/04/1982. MRC 061, 062, 066. Copiapó. Unknown collector. 19/07/1982. SSUC Re 322-26. Tierra Amarilla. J. Troncoso-Palacios & F. Ferri. cols. 23/11/2011. SSUC Re 330. Diego de Almagro. F. Ferri, J. Troncoso-Palacios cols. 09/12/2011. SSUC Re 327-29, 331-34, 419. Diego de Almagro. F. Ferri & J. Troncoso-Palacios cols. 12/12/2011.

*Liolaemus zapallarensis*. MZUC 29118. Las Tacas, Región de Coquimbo. J.C. Ortiz col. 15/10/1976. MZUC 29127. Las Tacas, Lagunillas, sector rocoso de la playa. J.C. Ortiz & J. Simonetti cols. 10/09/1977. SSUC Re 472. Totoralillo, Región de Coquimbo. J. Troncoso-Palacios & Y. Marambio cols. 12/12/2011.

## References

[B1] Bell T (1843) Reptiles. In: Darwin C (Ed.). The Zoology of the Voyage of the HMS Beagle, Under the Command of Captain Fitzroy, RN, During the Years 1832 to 1836.Smith, Elder and Co, London, Volume 5: 1-51

[B2] Boulenger GA (1885) Catalogue of the lizards in the British Museum (Natural History). Second edition, Volume 2. Taylor and Francis, London, xiii + 497 pp.

[B3] Domeyko I (1859) Publicaciones de algún interés hechas en Alemania y en Francia sobre la jeografia, jeología, historia natural e industria minera de América y especialmente de Chile. Anales de la Universidad de Chile 16: 426-469

[B4] Donoso-Barros R (1954) Consideraciones sobre la ecología de los reptiles del sur de Coquimbo. Zooiatría (Santiago) 3: 3-5

[B5] Donoso-Barros R (1966) Reptiles de Chile. Ediciones de la Universidad de Chile, Santiago, cxliv + 458 pp.

[B6] Donoso-Barros R (1975) Nuevos repiles y anfibios de Chile. Boletín de la Sociedad de Biología de Concepción 48: 217-229

[B7] Duméril AMC Bibron G (1837) Erpétologie genérale ou histoire naturelle complète des reptiles. Volume 4. Librarie Enclyclopedique de Roret, Paris, 571 pp.

[B8] Etheridge RE (1995) Redescription of *Ctenoblepharys adspersa* Tschudi, 1845, and the taxonomy of Liolaeminae (Reptilia: Squamata: Tropiduridae). American Museum Novitates 3142: 1-34

[B9] Fitzinger L (1843) Systema Reptilium, fasciculus primus, Amblyglossae. Braumüller et Seidel, Wien, 106 pp.

[B10] Girard CF (1858a) Abstract of a report to Lieut. James M. Gilliss, USN, upon the reptiles collected during the USN. Astronomical Expedition to Chili. Proceedings of the Academy of Natural Sciences of Philadelphia 7: 226-227

[B11] Girard CF (1858b) United States Exploring Expedition During the Years 1838, 1839, 1840, 1841, 1842, Under the Command of Charles Wilkes, USN. Vol. 20 (Herpetology). Lippincott JB, Philadelphia, XV + 492 pp.

[B12] Gray JE (1845) Catalogue of the specimens of lizards in the collection of the British Museum. Edward Newman, London, xxvii + 289 pp.

[B13] Hellmich W (1950) Die Eidechsen der Ausbeute Schröder (Gattung *Liolaemus*, Iguan.) (Beiträge zur Kenntnis der Herpetofauna Chiles XIII). Veröffentlichungen der Zoologischen Staatssammlung München 1: 129-194

[B14] ICZN (1999) International Code of Zoological Nomenclature. The International Trust for Zoological Nomenclature, London, xxiv + 306 pp.

[B15] Langstroth RP (2011) On the species identities of a complex *Liolaemus* fauna from the Altiplano and Atacama Desert: insights on *Liolaemus stolzmanni*, *L. reichei*, *L. jamesi pachecoi*, and *L. poconchilensis* (Squamata: Liolaemidae). Zootaxa 2809: 20-32

[B16] Laurent RF (1995) Sobre una pequeña colección de lagartos del género *Liolaemus* (Tropiduridae) proveniente del extremo suroeste de Bolivia. Cuadernos de Herpetología 9: 1-6

[B17] Lobo F (2001) A phylogenetic analysis of lizards of the *Liolaemus chiliensis* group (Iguania: Tropiduridae). Herpetological Journal 11: 137-150

[B18] Lobo F (2005) Las relaciones filogenéticas dentro grupo *chiliensis* (Iguania: Liolaemidae: *Liolaemus*): sumando nuevos caracteres y taxones. Acta Zoologica Lilloana 49: 65-87

[B19] Meyen FJF (1834) Reise um die erde ausgeführt auf dem Königlich preussischen seehandlungs-schiffe Prinzess Louise, commandirt von captain W. Wendt, in den jahren 1830, 1831 und 1832. Sander’sche buchhandlung, Berlin, Volume VIII, 494 pp.

[B20] Müller L Hellmich W (1933a). Beiträge zur Kenntnis der Herpetofauna Chiles. VI. Ueber einige *Liolaemus* Arten des Berliner Naturkundlichen Museums. Zoologischer Anzeiger 101: 121-134

[B21] Müller L Hellmich W (1933b) Beiträge zur Kenntnis der Herpetofauna Chiles. VII. Der Rassenkreis des *Liolaemus nigromaculatus*. Zoologischer Anzeiger 103: 128-142

[B22] Ortiz JC (1981) Estudio multivariado de las especies de *Liolaemus* del grupo *nigromaculatus* (Squamata, Iguanidae). Anales del Museo de Historia Natural de Valparaíso 14: 247-265

[B23] Ortiz JC (1987) Une nouvelle espèce de *Liolaemus* (Sauria, Iguanidae) du Chili. Bulletin du Museum National d’Histoire Naturelle (Paris). Section A, Zoologie, Biologie et Ecologie Animales 9: 265-270

[B24] Ortiz JC (1989) Description de *Liolaemus silvai* sp. nov. (Sauria, Iguanidae) du “Norte Chico” du Chili. Bulletin du Museum National d’Histoire Naturelle. Naturelle (Paris). Section A, Zoologie, Biologie et Ecologie Animales 11: 247-252

[B25] Ortiz JC Núñez H (1986) Catálogo crítico de los tipos de reptiles conservados en el Museo Nacional de Historia Natural, Santiago, Chile. Publicación Ocasional del Museo Nacional de Historia Natural 43: 3-23

[B26] Philippi RA (1860) Reise durch die Wüste Atacama, auf Befehl der chilenischen Regierung im Sommer 1853–1854. Eduard Anton, Halle, ix + 192 pp.

[B27] Pincheira-Donoso D Núñez H (2005) Las especies chilenas del género *Liolaemus* (Iguanidae Tropiduridae, Liolaeminae). Taxonomía, sistemática y evolución. Publicación Ocasional. Museo Nacional de Historia Natural, Santiago 59: 7-486

[B28] Shreve B (1941) Notes on Ecuadorian and Peruvian reptiles and amphibians with description of new forms. Proceedings of the New England Zoological Club 18: 71-83

[B29] Simonetti J Núñez H (1986) Sympatry and taxonomy of two lizards of the *Liolaemus nigromaculatus* group in northern Chile. Journal of Herpetology 20 (3): 474-475 doi: 10.2307/1564522

[B30] Steindachner F (1867) Reise der österreichischen Fregatte Novara um die Erde in den Jahren 1857, 1858, 1859 unter den Bafehlen des Commodore B. von Wüllerstorf-Urbair. Zologischer Theil. 3. Reptilien. Hof- und Staatsdruckerei KK, Wien, 98 pp.

[B31] Steindachner F (1891) Ueber die Reptilien und Batrachier der westlichen und oestlichen Gruppe der Kanarischen Inseln. Ann. K.K. Hofmuseums Wien 6: 287-313

[B32] Troncoso JF Ortiz JC (1987) Catálogo Herpetológico del Museo Regional de Concepción. Comunicaciones del Museo Regional de Concepción (Chile) 1: 9-19

[B33] Troncoso-Palacios J Etheridge R (2012) Distributional range of the poorly known *Liolaemus tacnae* (Shreve 1941). Herpetological Bulletin 121: 35-38

[B34] Troncoso-Palacios J Marambio Y (2011) Lista comentada de los reptiles de la Región de Atacama. Boletín del Museo Regional de Atacama (Copiapó) 2: 60-78

[B35] Uetz P (2012) The Reptile Database http://www.reptile-database.org (accessed Dec 31, 2012).

[B36] Valladares P (2011) Análisis, síntesis y evaluación de la literatura de lagartos de la Región de Atacama, Chile. Gayana 75: 81-98 doi: 10.4067/S0717-65382011000100006

[B37] Werner F (1898) Die Reptilien und Batrachier der Sammlung Plate. Zoologische Jahrbücher. Supplementheft. Jena 4: 244-278

[B38] Wiegmann AFA (1834) Beiträge zur Zoologie, gesammelt auf einer Reise um die Erde, von Dr. Meyen FJF, M.D.A.D.N. Siebente Abhandlung. Amphibien. Nova Acta Physico-medica Academiae Caesareae Leopoldino-Carolinae Naturae Curiosorum. Halle, 17: 183-268

